# Pre-clinical safety and therapeutic efficacy of a plant-based alkaloid in a human colon cancer xenograft model

**DOI:** 10.1038/s41420-022-00936-3

**Published:** 2022-03-28

**Authors:** Jessica L. Freeling, Jamie L. Scholl, Morgan Eikanger, Cole Knoblich, Rashaun A. Potts, David J. Anderson, Joseph E. Rower, Mohammad Hadi Farjoo, Haotian Zhao, Angela Pillatzki, Khosrow Rezvani

**Affiliations:** 1 Division of Basic Biomedical Sciences, Physiology Core Facility, The University of South Dakota, Vermillion, SD USA; 2 Division of Basic Biomedical Sciences, Center for Brain and Behavior Research, The University of South Dakota, Vermillion, SD USA; 3 Division of Basic Biomedical Sciences, Sanford School of Medicine, The University of South Dakota, Vermillion, SD USA; 4 Center for Human Toxicology, University of Utah, Salt Lake City, UT USA; 5 Department of Pharmacology, School of Medicine, Shahid Beheshti University of Medical Sciences, Tehran, Iran; 6 New York Institute of Technology, College of Osteopathic Medicine, Old Westbury, NY USA; 7 Veterinary and Biomedical Sciences Department, Animal Disease Research and Diagnostic Laboratory, South Dakota State University, Brookings, SD USA

**Keywords:** Drug development, Drug safety

## Abstract

A high-throughput drug screen revealed that veratridine (VTD), a natural plant alkaloid, induces expression of the anti-cancer protein UBXN2A in colon cancer cells. UBXN2A suppresses mortalin, a heat shock protein, with dominant roles in cancer development including epithelial–mesenchymal transition (EMT), cancer cell stemness, drug resistance, and apoptosis. VTD-dependent expression of UBXN2A leads to the deactivation of mortalin in colon cancer cells, making VTD a potential targeted therapy in malignant tumors with high levels of mortalin. VTD was used clinically for the treatment of hypertension in decades past. However, the discovery of newer antihypertensive drugs and concerns over potential neuro- and cardiotoxicity ended the use of VTD for this purpose. The current study aims to determine the safety and efficacy of VTD at doses sufficient to induce UBXN2A expression in a mouse model. A set of flow-cytometry experiments confirmed that VTD induces both early and late apoptosis in a dose-dependent manner. In vivo intraperitoneal (IP) administration of VTD at 0.1 mg/kg every other day (QOD) for 4 weeks effectively induced expression of UBXN2A in the small and large intestines of mice. Liquid chromatography–tandem mass spectrometry (LC–MS/MS) assays on tissues collected from VTD-treated animals demonstrated VTD concentrations in the low pg/mg range. To address concerns regarding neuro- and cardiotoxicity, a comprehensive set of behavioral and cardiovascular assessments performed on C57BL/6NHsd mice revealed that VTD generates no detectable neurotoxicity or cardiotoxicity in animals receiving 0.1 mg/kg VTD QOD for 30 days. Finally, mouse xenograft experiments in athymic nude mice showed that VTD can suppress tumor growth. The main causes for the failure of experimental oncologic drug candidates are lack of sufficient safety and efficacy. The results achieved in this study support the potential utility of VTD as a safe and efficacious anti-cancer molecule.

## Introduction

Colorectal cancer (CRC) is a major cause of cancer deaths globally [1]. Since 2000, the rate of CRC in adults under fifty years of age has dropped due to improved screening. However, declines in overall CRC incidence have been masked by the increasing occurrence of CRC in younger adults who are not included in current cancer screening recommendations [2]. Despite advances in screening strategies and treatment regimens, 50% of CRC patients develop recurrent disease with a poor overall 5-year survival rate [3]. Thus, a major challenge is the development of novel targeted therapies, particularly for metastatic forms of the disease.

We discovered that the UBX-domain-containing protein UBXN2A [4] binds to and inactivates the protein mortalin, an oncoprotein known to occur in several kinds of solid tumors, including CRC [5]. A high-throughput drug screen followed by biochemical analysis showed that veratridine (VTD), an alkaloid derived from Liliaceae plants, induces UBXN2A, leading to suppression of mortalin and reactivation of WT-p53, a tumor suppressor protein [6]. Our previous work highlighted that p53 is an important positive modulator in the UBXN2A-mortalin pathway but that it is not an essential core component of UBXN2A’s anti-cancer pathway [6]. Additionally, VTD reduces colony formation in HCT-116 p53+/− or p53−/−. Among patients with colon tumors, half have overexpressed mortalin in comparison to adjacent normal tissues [6]. This overexpressed mortalin correlates with poor survival rates [7]. Despite mortalin’s clear role in tumorigenesis, direct mortalin inhibition by small molecules was previously unsuccessful in clinical trials due to kidney failure [8]. However, UBXN2A-dependent inhibition of mortalin in the presence of a safe and tolerable dose of VTD offers a colon cancer-specific treatment option.

VTD, as an activator of voltage-dependent Na+ channels [9, 10], induces toxicity in both neuronal cultures [11, 12] and hippocampal slices [12]. Administration of VTD by the intraperitoneal (IP) route to rats induces neurotoxic effects such as wet dog shake behavior and apoptosis in the rat hippocampus [13]. Complete protection against VTD-induced neurotoxicity is possible by blocking VTD-sensitive Na+ channels [14, 15]. Additionally, it has been reported that VTD can produce a toxic effect on the heart in an isolated left atrial and aortic model of the rat [13, 16]. However, it is unknown whether the lower dosage of VTD necessary to induce expression of UBXN2A exhibits any toxic effects. Therefore, the objective of this research is to study the expression, safety, and efficacy of VTD in both in vitro and animal models. We hypothesize that IP injection of VTD at 0.1 mg/kg every other day (QOD), a dose 10 times lower than the known LD50, is sufficient to induce UBXN2A expression and will exhibit no neurotoxicity or cardiotoxicity in an animal model. This study will reveal a novel platform for a tolerable and safe anti-mortalin drug for patients with CRC.

## Results

### Veratridine induces early and late apoptosis in colon cancer cells

**Fig. 1:**
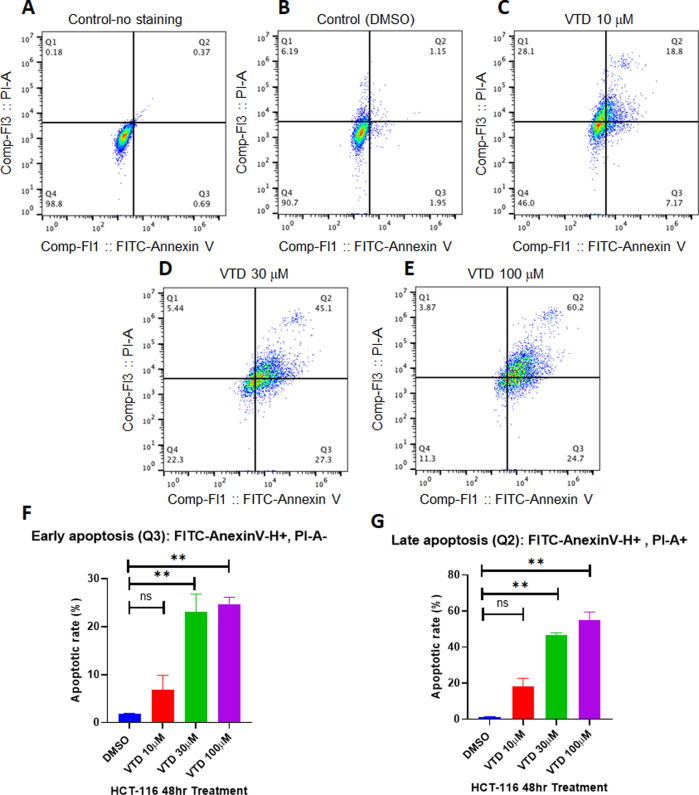
Veratridine induces early and late apoptosis in colon cancer cells. **A**–**E** HCT-116 colon cancer cells were treated with VTD or DMSO for 48 h. Flow-cytometry experiments followed by FlowJo software analysis revealed that VTD significantly increases Annexin V-positive and Annexin V/PI-positive cell populations in a dose-dependent manner. **F**, **G** Graphical representation of flow-cytometry results in early and late apoptosis performed in triplicate and expressed as the mean ± standard deviation (**P < 0.01).

Our previously published results showed that VTD can suppress cell proliferation and induce apoptosis mediated by the UBXN2A–mortalin–p53 axis [6]. Effective pro-apoptotic agents have shown strong potential value in cancer drug discovery [17], particularly natural alkaloid products [18, 19]. We determined whether the incubation of cancer cells with VTD can induce both early and late apoptosis in HCT-116 human colon cancer cells. Figure 1 shows that a significant population of HCT-116 cells enter early and late apoptosis in the presence of VTD. VTD showed a dose-dependent manner effect on late apoptosis (Fig. 1G). The mean percentages of early apoptosis are as follows: DMSO (1.8%), VTD 10 μM (6.89%), VTD 30 μM (23%), and VTD 100 μM (24.7%), with a *P*-value of <0.01. The mean percentages of late apoptosis are as follows: DMSO (1.24%), VTD 10 μM (18.06%), VTD 30 μM (46.5%), and VTD 100 μM (54.76%), with a *P*-value of <0.01. The EC50 values of VTD are likely in the micromolar range [20] which can be suboptimal for drug development and may cause off-target toxicity at the cellular level. The examined concentrations (10, 30, and 100 μM) used in Fig. 1 is based on two previous studies: (1) Jordan et al. showed 30 μM VTD effectively induces apoptosis in cells [21] and (2) Weiser et al. reported that 100 μM veratridine is not toxic to the cells [22]. The presented results in Fig. 1F, G clearly show that VTD as low as 30 μM can induce a significant early and late apoptosis in vitro. We found similar but not significant apoptosis with 10 μM VTD. This provides further evidence of the effective activation of apoptotic pathways induced by VTD in cancer cells and a comprehensive rationale for the development of VTD as a potential cancer therapy by targeting apoptosis pathways.

### Veratridine exhibits no outward neurotoxicity at 0.1 mg/kg

The LD_50_ (lethal dose 50%) of VTD is 1.35 and 4.9 mg/kg for mice injected intraperitoneally and subcutaneously, respectively [23, 24]. Considering this, we conducted a maximum tolerated dose (MTD) experiment and administered VTD IP at 0.1, 0.3, 0.5, and 1 mg/kg to C57BL/6NHsd mice. Animals were visually monitored for VTD-described neurotoxicity signs and symptoms based on previous reports [16]. As reported by Otoom et al. [13] we observed dose-dependent signs of neurotoxicity with increasing VTD dosages. At the lowest VTD dose of 0.1 mg/kg, no outward neurological symptoms were noted. At 0.3 mg/kg, a brief frozen state lasting less than one minute was observed. At 0.5 mg/kg, animals exhibited a frozen state that lasted several minutes and then resolved. At 1 mg/kg, the animals exhibited severe freezing and respiratory distress and thus were immediately euthanized. The absence of neurotoxic signs at 0.1 mg/kg is similar to results previously reported in rats by Meilman et al. [13, 25].

### Veratridine induces expression of UBXN2A in vivo

**Fig. 2:**
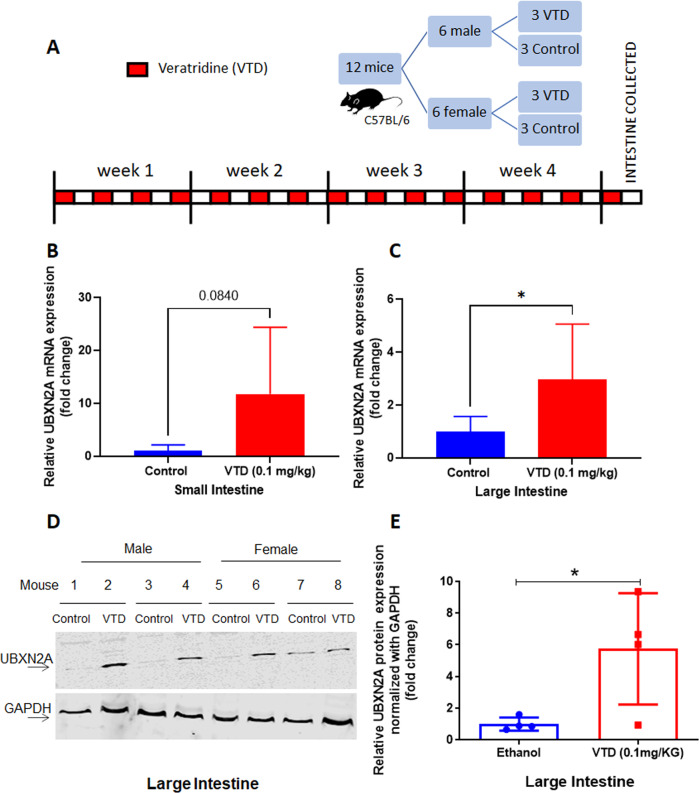
Veratridine induces expression of UBXN2A in vivo. **A** Mice were treated with control (ethanol 0.01%) or 0.1 mg/kg VTD QOD IP for 30 days, and intestine was collected at endpoint. **B**, **C** Extracted small and large intestine tissues were subjected to qRT-PCR. Significant elevation of UBXN2A RNA in response to VTD treatment (*N* = 6 per treatment, *P < 0.05) occurred in the large intestine. The small intestine showed a non-significant elevation of UBXN2A in response to VTD (*N* = 6 per treatment, *P* = 0.08). **D**, **E** Tissue lysates of the large intestine from 4 control and 4 VTD treated mice were subjected to WB (Panel **D**). VTD treatment significantly enhanced the protein level of UBXN2A in large intestine tissues, confirming the three-fold increased UBXN2A RNA level in the presence of VTD (Panel **E**, *N* = 4 per treatment, dots represent individual animal expression, *P < 0.05).

Based on the MTD results, we decided to examine whether 0.1 mg/kg of VTD can induce expression of UBXN2A in an animal model, which previously had only been shown at the cellular level [6]. To test this, C57BL/6NHsd mice were administered either VTD 0.1 mg/kg IP QOD or ethanol (0.01%) control. After 30 days, small and large intestinal tissues were collected for RNA and protein studies (Fig. 2A). The results in Fig. 2 show that treatment with VTD significantly induces expression of UBXN2A in mouse large intestine tissues in both RNA (Fig. 2C, mean control = 1, mean VTD = 2.97, *N* = 6 per treatment, *P* < 0.05) and protein (Fig. 2D, E, *P* < 0.05, *N* = 4 per treatment) compared to controls that received ethanol vehicle. The qRT-PCR showed that VTD increases the RNA level of UBXN2A in the small intestine but failed to attain statistical significance (Fig. 2B, mean control = 1, mean VTD = 11.77, *N* = 6 per treatment, *P* = 0.0840). Interestingly, qRT-PCR revealed that the large intestine section, which includes the descending section of the mouse colon, has a more uniform response to VTD. Consequently, western blot (WB) experiments confirmed significant translation of UBXN2A RNA to protein in response to VTD (Fig. 2E and Table 2, Supplemental Materials). We observed differences in response to VTD treatment among mice which could be due to variability in drug delivery/metabolism per animal. Additionally, the morphological heterogeneity of colon tissues and non-linear nature of WB signals across samples [26] could be two additional reasons for these differences. We used GAPDH to normalize the UBXN2A signal to show the level of UBXN2A per mouse. The results clearly show heterogeneity in the distal colon in individual mice. Further studies and alternative techniques can determine reasons behind these differences. Testing the low dose of VTD in an animal model confirmed that 0.1 mg/kg VTD can effectively elevate the level of UBXN2A in mouse colon tissues.

### Tissue bioanalysis shows no major accumulation of VTD in an animal model

**Fig. 3:**
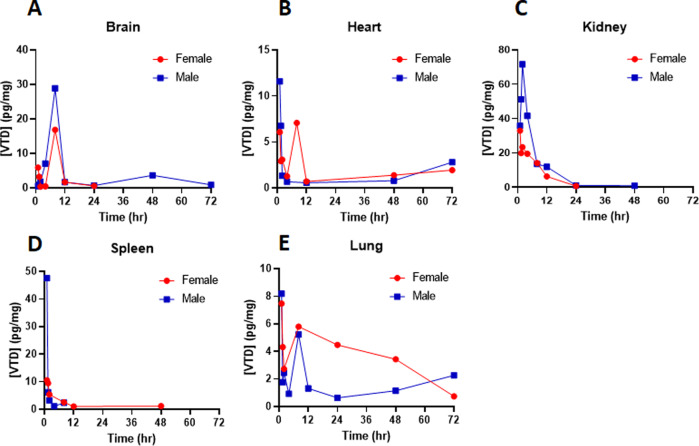
Tissue bioanalysis shows no major accumulation of VTD in an animal model. Mice were treated with a single dose of 0.1 mg/kg VTD followed by tissue extraction at timepoints of 1, 1.5, 2, 4, 8, 12, 24, 48, and 72 h. **A**–**E** Bioanalysis revealed that VTD concentrations were in the pg/mg tissue range in all collected tissues. VTD concentrations peaked at ~8 h at ~20–30 pg/mg but were largely eliminated by 12 h in brain tissues (**A**). VTD concentrations in heart tissue peaked immediately after IP injection but were rapidly eliminated (**B**). Similar findings were observed for kidney (**C**) and spleen (**D**) tissues. The status of VTD level in lung tissues suggests a possible secondary absorption mechanism (**E**). No VTD was detected in the liver, suggesting that VTD is largely renally cleared unchanged.

Plasma and tissue concentrations of VTD were determined by LC–MS/MS at 1, 1.5, 2, 4, 8, 12, 24, 48, and 72 h. Plasma concentrations were negligible (<0.20 ng/ml) for all tested samples, suggesting a minimal systemic presence of VTD upon low-dose administration in mice. Tissue concentrations overall were low, with only pg/mg concentrations in all studied tissues. VTD concentrations in brain tissue peaked at 8 h but were rapidly eliminated (Fig. 3A). Heart concentrations peaked immediately and were eliminated by 12 h (Fig. 3B), and all other samples showed drug elimination by 72 h (Fig. 3C–E). The low, if not undetectable, VTD concentrations at 48 h suggest that there is a negligible risk for drug accumulation with chronic dosing in all tissues except the lung. The lung is known to be naturally permeable to all small-molecule drugs [27]. Further studies will be conducted on lungs in the future. The clearance of VTD in this current study matches in vitro metabolic assays in rat liver microsomes [28].

### Acute and repeated exposure to veratridine does not impact behavior in mice

**Fig. 4:**
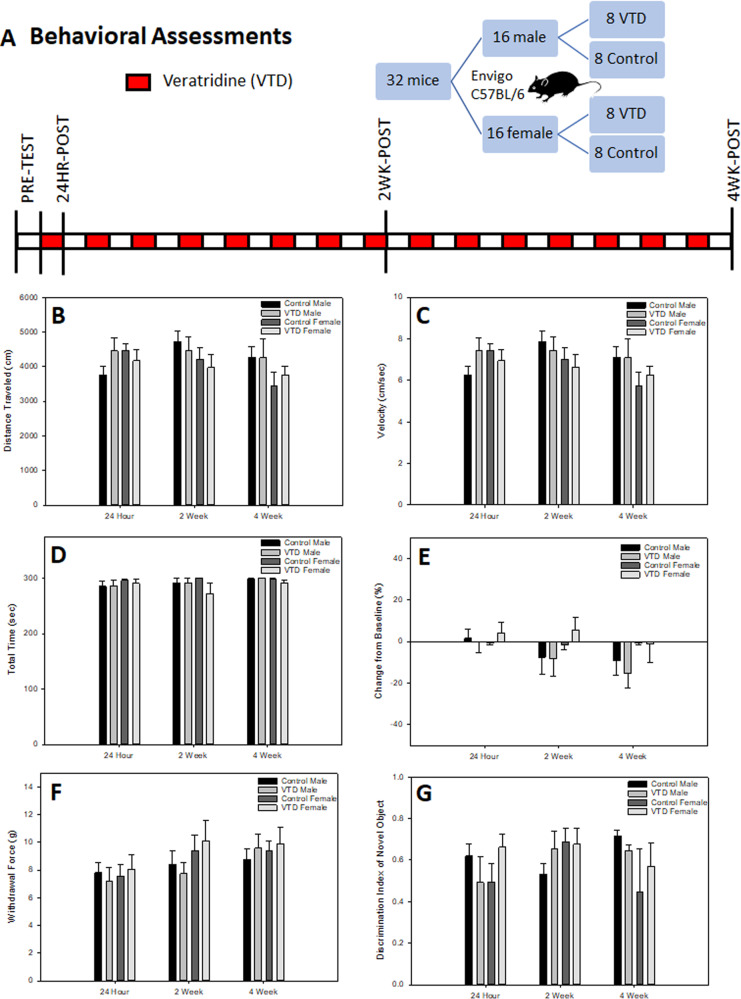
Acute and repeated exposure to VTD does not impact behavior in mice. **A** Experimental timeline for mice treated with control or 0.1 mg/kg VTD QOD IP for 30 days and evaluated for behavioral impacts. **B**, **C** Results from an open field test to evaluate locomotor behavior following acute and repeated exposure to VTD. No treatment effects were found in total distance traveled (Panel **B**) or overall velocity (Panel **C**) within the open field chamber. Results are expressed as mean ± SEM (*N* = 8 per group, *P* > 0.05 for all comparisons). **D**, **E** Results from rotarod and grid hang grip tests evaluating motor coordination, balance, and limb strength following acute and repeated exposure to VTD. No treatment effects were found in total time on rotarod (Panel **D**) or grid hang grip strength as expressed as a percent of baseline (Panel **E**). Results are expressed as mean ± SEM (*N* = 8 per group, *P* > 0.05 for all comparisons). **F**, **G** Results from mechanical nociception response evaluating pain threshold and novel object recognition evaluating short-term memory behavior following acute and repeated exposure to VTD. No treatment effects were found in the mechanical nociception response (Panel **F**) or short-term memory behavior exhibited as the discrimination index of a novel object (Panel **G**). Results are expressed as mean ± SEM (*N* = 8 per group, *P* > 0.05 for all comparisons).

The timeline for the behavioral cohort is shown in Fig. 4A. No significant differences between VTD and the control group were found in any behavioral measure (Table 1, Supplemental Materials). Locomotor testing revealed no effect of VTD within distance traveled (Fig. 4B) or velocity (Fig. 4C). Significant interactions were found between sex and time point in both distance traveled (*F*_[3,84]_ = 6.429; *P* = 0.001) and velocity (*F*_[3,84]_ = 6.403; *P* = 0.001), with females exhibiting greater exploratory behavior compared to males. This effect was expected, as females traditionally exhibit increased locomotor exploration compared to males in all forms of locomotor-based testing [29]. Post hoc analysis revealed decreased distance traveled and decreased velocity between baseline and the other time points in all groups as well as at the 4-week time point in females. No effect of VTD was found on measures of motor coordination (Fig. 4D), strength (Fig. 4E), or nociception (Fig. 4F). A significant effect of time point was found by rotarod testing (*F*_(1.609,45.055)_ = 45.378; *P* < 0.001), with post hoc analysis revealing training improvement from baseline to the other 3 time points. Training effects were also seen with the grid hang test (*F*_[3,84]_ = 6.046; *P* = 0.004), with mice jumping from the grid at the 4-week time point in all groups; and with Von Frey testing (*F*_[3,84]_ = 11.619; *P* < 0.001), with all mice habituating to the stimuli at both the 2-week and 4-week time points compared to baseline. No significant differences were found in any measure in novel object recognition testing (Fig. 4G).

### Veratridine has no impact on cardiac function and exhibits a mild blood pressure-lowering effect

**Fig. 5:**
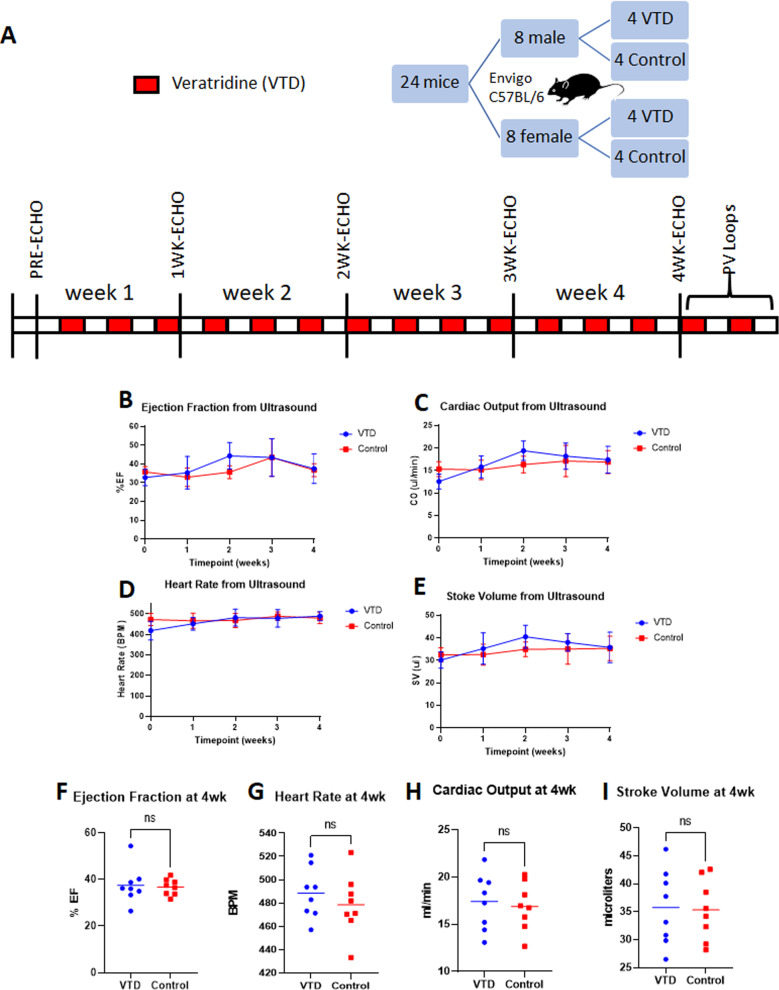
Echocardiograms reveal no impact of VTD on cardiovascular function. **A** Timeline of assessment of cardiovascular function using M-mode ultrasound throughout treatment with 0.1 mg/kg VTD QOD IP for 30 days. **B**–**E** Results from echocardiograms evaluating ejection fraction (Panel **B**), cardiac output (Panel **C**), heart rate (Panel **D**), and stroke volume (Panel **E**) showed no significant difference at any time point between VTD and control groups. Results are expressed as mean ± SEM (*N* = 8 per group, *P* > 0.05). **F**–**I** After 4 weeks of VTD treatment, no significant difference was noted for any parameter. Results are expressed as mean ± SEM (*N* = 8 per group, *P* > 0.05).

The timeline for the cardiovascular assessments is represented in Fig. 5A. Ejection fraction (EF) from ultrasound indicated no difference at any time point between VTD and control animals (Fig. 5B). Furthermore, no difference at any time point was detected for cardiac output (CO, Fig. 5C), heart rate (HR, Fig. 5D), or stroke volume (SV, Fig. 5E). After 4 weeks of drug treatment, there was no significant difference in any of the echo parameters by *t*-test (Fig. 5F–I) and no effect of sex by two-way ANOVA on HR or EF (data not shown). A sex difference was noted in CO (*F*_[1,12]_ = 9.906; *P* = 0.008) and SV (*F*_[1,12]_ = 11.95; *P* = 0.005) by two-way ANOVA, but there was no effect of treatment. Sex difference in CO and SV is common in C57BL/6NHsd mice due to the size difference between the sexes.

**Fig. 6:**
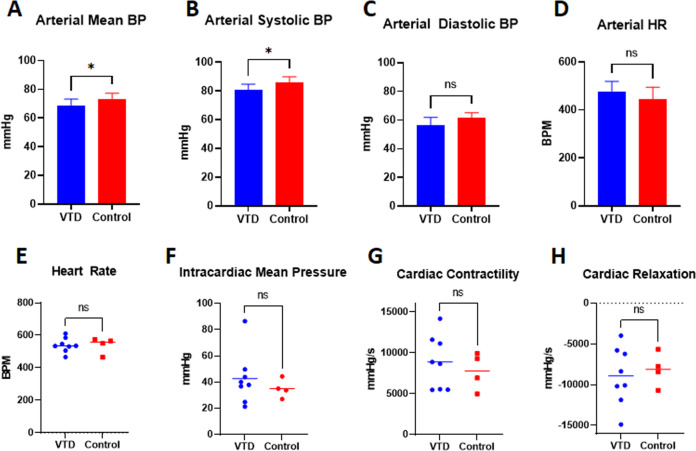
Cardiovascular hemodynamic assessments reveal a mild reduction in mean blood pressure. Arterial and intracardiac hemodynamic assessments were conducted at endpoint after treatment with 0.1 mg/kg VTD QOD for 30 days. **A**–**E** Carotid arterial blood pressure at the 4-week endpoint revealed a significant but mild decrease in arterial mean blood pressure (Panel A) with VTD treatment. This was due to a reduction in arterial systolic (Panel **B**) but not diastolic (Panel **C**) blood pressure. Arterial heart rate was unaffected (Panel **D**). Results are expressed as mean ± SEM (*N* = 4–8 per group, *P < 0.05). **E**–**H** Intracardiac pressure-volume loop (PV loop) analysis at the 4-week endpoint revealed no significant difference for heart rate (Panel **E**), mean pressure (Panel **F**), contractility (Panel **G**), or relaxation (Panel **H**). Results are expressed as mean ± SEM (*N* = 4–8/grp, *P* > 0.05).

Because of the previous use of VTD as an anti-hypertensive and because echocardiograms do not enable blood pressure evaluation, we additionally recorded hemodynamic data at endpoint. Recording of carotid arterial blood pressure after 4 weeks of VTD treatment showed a significant, but physiologically mild, decrease in arterial mean blood pressure (mBP) in VTD versus control animals of 4.93 ± 2.24 mmHg, *P* = 0.047 (Fig. 6A). Further evaluation revealed that specifically systolic blood pressure (sBP), *P* = 0.025, but not diastolic blood pressure (dBP), *P* = 0.073, was impacted (Fig. 6B, C) as previously described [25]. As was shown with echocardiogram data, HR was not impacted by treatment (Fig. 6D). Finally, two-way ANOVA indicated no sex differences with treatment.

As with HR from echocardiogram and arterial measurements, intracardiac HR also showed no difference with VTD treatment (Fig. 6E). Unlike arterial mean pressure, however, there was no significant difference between treatment groups in intracardiac mean pressure (Fig. 6F). Furthermore, no differences in d*p*/d*t* max or d*p*/d*t* min, contractility and relaxation, respectively (Fig. 6G, H), were observed.

### Veratridine reduces tumor size

**Fig. 7:**
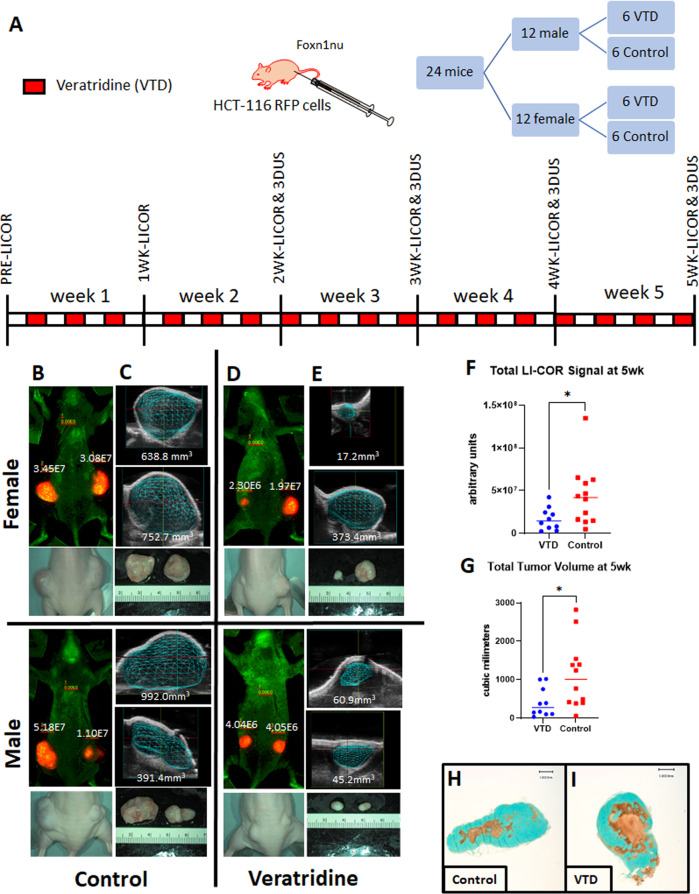
VTD significantly slows tumor growth in a xenograft mouse model of colon cancer. **A** Mice were inoculated subcutaneously into both flanks with 1 × 10^6^ iRFP-HCT-116 cells and treated with VTD 0.1 mg/kg QOD or control using the depicted timeline. **B**, **E** iRFP signals and volume of developed xenograft tumors measured by LI-COR (Panels **B**, **D**) and 3D ultrasound (Panels **C**, **E**) at the 5-week endpoint. **F**, **G** VTD significantly decreased the iRFP signal in xenograft tumors measured by LI-COR technology (**F**). Tumor size was significantly decreased in VTD treated mice after 5 weeks as measured by 3D ultrasound (**G**) (*N* = 10–12/grp, *P < 0.05). **H**, **I** Representative tumor tissue stained by TUNEL assay suggests that VTD-treated xenograft tumors had more apoptotic and necrotic tissues.

Figure 7 presents data from the in vivo mouse xenograft model. Foxn1nu mice were subcutaneously implanted with iRFP-tagged HCT-116 colorectal cancer cells (Table 3, Supplemental Materials) and subsequently treated with 0.1 mg/kg VTD IP QOD or control beginning one day after tumor implantation (Fig. 7A). 3D ultrasound to calculate precise tumor volume followed by LI-COR near-infrared imaging to visualize the effects of VTD on primary tumor growth was conducted weekly over 5 weeks (Fig. 7B–E and Supplemental Fig. S1). After 5 weeks, VTD treatment resulted in a significant reduction of iRFP signal, with a mean total LI-COR signal in the VTD group of 1.68E7 a.u. and in the control group of 4.37E7 a.u., *P* = 0.035 (Fig. 7F). Tumor volume was also significantly reduced, with mean total tumor volume in the VTD group of 409.3 mm^3^ and in the control group of 1117 mm^3^, *P* = 0.028 (Fig. 7G). As previously shown at the cellular level [30], the presence of VTD led to the induction of apoptotic and necrotic tumors. The TUNEL staining of extracted xenograft tumors indicates that VTD induces cell death in tumor tissues with larger regions of apoptotic/necrotic tissues in mice treated with VTD (Fig. 7H, I). A set of WB experiments (Supplemental Fig. S2) confirmed VTD treatment increases protein level of UBXN2A while decreases the level of mortalin indicating the negative regulatory role of UBXN2A on mortalin. These data show that 0.1 mg/kg VTD QOD can effectively function as an anti-growth agent.

## Discussion

**Fig. 8:**
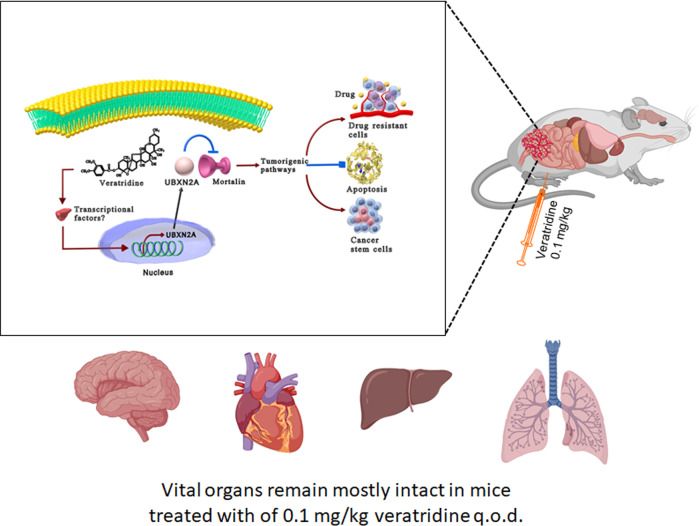
Diagrammatic representation of anti-cancer mechanism of action of VTD. This schematic shows the potential anti-tumorigenic function of VTD in a xenograft mouse model of human cancer created with Biorender.com. A 0.1 mg/kg dose of VTD increases transcription and protein expression of UBXN2A in human cancer cells originated from the epithelial cells lining the colon. Enhanced UBXN2A binds to a well-studied oncoprotein, mortalin, and suppresses mortalin’s tumorigenic functions. Mounting evidence indicates that the inhibition of mortalin leads to cell apoptosis, suppression of cancer stem cells (CSCs), and reduction of drug resistance. Meanwhile, VTD showed no significant toxicity toward vital organs, such as the heart, brain, liver, or lung. Further studies in CRC animal models will pave the way to clinically translate the use of VTD for CRC patients in the near future.

The need for alternative targeted and adjuvant therapies has become more important because of the increased incidence of CRC among younger patients and the lack of effective drugs for metastatic forms of CRC [31]. Mortalin, an oncoprotein with several partners, is involved in multiple cellular pathways that facilitate tumor growth and metastasis such as triggering innate tumor-suppressive mechanisms, supporting angiogenesis, protecting cancer stem cells, and drug resistance [32]. Evidence in the literature [7, 33–35] plus our published data [4, 6] indicates that targeting mortalin oncoprotein offers significant therapeutic benefit in CRC and could advance clinical improvement in patients. The potential role of mortalin oncoprotein as a therapeutic target has been underscored since 1998 [36]. Nevertheless, to date, no successful FDA-approved drug has been achieved for inhibiting mortalin’s tumorigenic role in patients. A recent report has shown that Mortaparib^Plus^, a novel synthetic small-molecule triazole derivative, interferes with mortalin–p53 interaction, resulting in suppression of cell proliferation in situ [37]. However, the anti-cancer mechanism of Mortaparib^Plus^ needs future validation in both animal models and clinical settings. Another small molecule, MKT-077, was reported as a mortalin inhibitor with anti-cancer mechanisms [38]. However, MKT-007 triggered several unacceptable side effects, including renal damage, which led researchers to abort the clinical trial [39]. Our results reveal that VTD exhibits no such toxicity, yet it can precisely target mortalin (Fig. 8). The in vivo concentrations of VTD measured in mice tissue are approximately an order of magnitude lower than that the tested in vitro concentrations. It is notable, however, that the tissues studied in vivo were targeted due to their potential role in veratridine-associated toxicity, and not their role in VTD efficacy. It is unknown what VTD concentration is required in blood (or tumor tissue) to reduce tumor size via the UBX2NA–mortalin axis. Addressing this question will require further work that is outside the scope of this manuscript.

Experimental small molecule oncology therapeutics have generally low success rates for entry into and completion of clinical trials. The major factors behind the failure of oncologic drugs in the last two decades can largely be attributed to a lack of clinical safety and efficacy [36]. One way to strike a balance between efficacy and safety is by the employment of adjuvant therapies that can enhance the action of, or enable the reduction of, chemotherapeutic dose. Targeted cancer therapy in CRC can be a potent strategy when combined with chemotherapy as a second-line treatment [40]. An example of a successful FDA-approved anti-cancer plant alkaloid is the semi-synthetic vinca alkaloid vinflunine [41, 42]. Thus, current evidence supports the potential benefit and drug ability of natural plant molecules in different types of malignancy [43, 44].

In our previous publication, we used a high-throughput drug screen to identify that VTD functions as a potent UBXN2A enhancer at cellular levels [6]. In this study, we confirm that VTD-dependent expression of UBXN2A induces apoptosis in cancer cells, effectively exploiting a major anti-cancer mechanism with a small molecule [17]. We show that VTD increases UBXN2A RNA and protein levels in mouse large intestines at a tolerable dose of 0.1 mg/kg. While VTD is known to cross the blood–brain barrier [13], our bioanalysis results show that VTD at 0.1 mg/kg is rapidly eliminated and has no long-lasting effects systemically. VTD at 0.1 mg/kg does not accumulate in vital organs, including the brain and heart. Furthermore, thorough behavioral and cardiovascular assessments indicate that VTD results in no toxicity at the 0.1 mg/kg dose. Finally, treatment with VTD in HCT-116 tumor-bearing athymic nude mice effectively reduces tumor growth and induces cell death (Fig. 8). Therefore, we conclude that our optimized dose of VTD is a selective and innovative tool for studying the impact of UBXN2A induction in a mouse model, accomplished at low doses and without toxicity. Indeed, a combined therapy using VTD and a reduced dose of chemotherapy drugs could further control tumor growth while slowing the development of drug resistance enhanced by mortalin. The past usage of VTD as an anti-hypertensive in humans [25] combined with the pre-clinical results in the present study represents a prospective repurposing strategy for VTD. There is strong potential to turn VTD into a safe and effective targeted therapy in CRC as has been accomplished with several other natural plant products [45]. Taken together, the safety and efficacy results presented here validate the utility of VTD as a targeted therapy not only in CRC [35, 46] but also as a potential targeted therapy in other solid tumors with high levels of mortalin, including breast cancer [47], hepatocellular carcinoma [48] and ovarian cancer [49].

## Materials and methods

### Animals and VTD treatment

Five different in vivo assessments were performed: maximum tolerated dose (MTD), behavioral, cardiovascular, blood/tissue concentration, and xenograft. C57BL/6NHsd mice were used for the MTD, behavioral, cardiovascular, and blood/tissue cohorts, and Foxn1nu athymic nude mice were used for the xenograft cohort. All mice were obtained from Envigo (Denver, CO, USA). For each type of assessment, separate cohorts of mice were utilized; however, when possible and appropriate, animal data from cohorts were combined to reduce animal numbers needed. Animal numbers utilized for each set of experiments are included with their associated figure legends.

All mice used in this study were maintained in the Animal Resource Center at the University of South Dakota, housed with 3–4 animals per cage, kept at 22 °C on a standard light cycle (lights on from 10:00 to 22:00 h), and provided free access to water and standard rodent chow. All experimental procedures were approved by the Institutional Animal Care and Use Committee of the University of South Dakota, in accordance with federal guidelines. All animal treatments and group assignments were randomized. Institutionally supported core facilities performed comprehensive assessments of the impact of VTD on live animals. Both the Physiology Core Facility [50–53] and the Behavioral Core Facility [29, 54, 55] at the University of South Dakota have published in their respective specialties. Blood/tissues were collected, and subsequent bioanalysis was performed by collaborators at the Center for Human Toxicology at the University of Utah. All data were collected, measured, and statistically analyzed by individuals within each facility who were blinded to animal treatment. Details about statistical methods used for each of the behavioral, cardiovascular, xenograft, and laboratory assessments can be found in Supplemental Materials.

In the behavioral, cardiovascular, and xenograft cohorts, drug concentration was given to match the dose required to induce UBXN2A expression from previous experiments and the timeline/frequency of typical cancer treatment paradigms. The dose of VTD was given based on mean weekly body weight (BW) by sex, and vehicle control was matched by volume.

In the cardiovascular and behavioral cohorts, the mean BW of males was 26.7 ± 4.5 g and of females was 20.9 ± 2.3 g at the 4-week endpoint. In the xenograft cohort at the 5-week endpoint, the mean BW of males and females was 31.3 ± 2.6 and 26.2 ± 1.9 g, respectively. All animals were weighed at weekly intervals and monitored carefully for health. In the cardiovascular and behavioral cohorts, none of the mice exhibited weight loss at any weighing interval, and all were alive and healthy at the ~4-week endpoint. Some of the xenograft animals exhibited minor weight loss once tumors became large during the 5th week. However, all animals were active and hydrated and remained in the study until endpoint.

### Maximum tolerated dose (MTD) cohort

In the MTD cohort, we conducted a MTD experiment and administered VTD IP at 0.1, 0.3, 0.5, and 1 mg/kg to C57BL/6NHsd mice. Animals were visually monitored for any signs or symptoms of neurotoxicity.

### Blood/tissue cohort

In the blood/tissue cohort, VTD at 0.1 mg/kg was given by the IP route. One male and one female C57BL/6NHsd mouse aged 12–16 weeks was sacrificed at each timepoint for later bioanalysis. The timepoints were 1, 1.5, 2, 4, 8, 12, 24, 48, and 72 h after IP injection of 0.1 mg/kg VTD. Animals were anesthetized with isoflurane to effect and administered 10 µl of 1000IU heparin via retro-orbital intravenous injection just before euthanasia to enable the collection of unclotted blood by cardiac stick. Blood was collected and spun at 1000×*g* for 3 min. Plasma and tissues were collected and frozen for later bioanalysis. Tissues collected included brain, heart, kidney, spleen, and lung. Details about the bioanalyses performed can be found in Supplemental Materials.

### Behavioral and cardiovascular cohorts

Mice in the behavioral and cardiovascular cohorts received VTD 0.1 mg/kg IP QOD for a total of 30 days. For the cardiovascular and behavioral cohorts, both male and female C57BL/6NHsd mice at 8–12 weeks of age were randomized into VTD and control groups. For the behavioral cohort, N = 16 mice were randomized to each of the treatment groups, with equal numbers of mice for each sex. Behavioral assessments encompassed a comprehensive battery of tests to evaluate motor coordination and balance, limb strength, sensory and pain threshold, and working memory in mice during VTD treatment. For the cardiovascular cohort, *N* = 8 mice were randomized to each of the VTD and control groups, with equal numbers of each sex. Cardiovascular assessments involved weekly evaluation of cardiac function using echocardiogram followed by endpoint evaluation of arterial and intracardiac hemodynamics. A timeline of the behavioral and cardiovascular assessments can be found with their respective results figures. Detailed descriptions of the evaluations performed can be found in Supplemental Materials.

### Xenograft cohort

For the xenograft cohort, *N* = 12 male and female athymic nude-Foxn1nu mice at 7–8 weeks of age were randomized into each of the VTD and control groups. Mice received bilateral injections of 1 × 10^6^ iRFP-tagged HCT-116 colorectal cancer cells suspended in 200 μl Hanks’ buffer free-FBS in the subcutaneous space over each hindquarter to induce tumors. Mice were treated with VTD 0.1 mg/kg IP QOD for a total of 37 days beginning the day after tumor induction. The formation and progression of tumors were monitored weekly with near-infrared fluorescent imaging using a LI-COR Classic Imager with MousePOD accessory (LI-COR Biosciences, Lincoln, NE, USA) and 3D ultrasound volume reconstructions using high-frequency ultrasound weekly beginning at 2 weeks. Tissues were collected at endpoint for downstream immunohistochemistry and biological experiments. A detailed description of the performed methods including biological and histological techniques can be found in Supplemental Materials.

## Supplementary information

Supplemental text

Supplemental Fig. 1

Supplemental Fig. 2

The original WB images

## Data Availability

The original contributions presented in the study are included in the article under Supplemental Materials; further inquiries can be directed to the corresponding author.
