# Supplementary material for: Pre-clinical safety and therapeutic efficacy of a plant-based alkaloid in a human colon cancer xenograft model
Source: Cell Death Discov. 2022 Mar 28;8:135. doi: 10.1038/s41420-022-00936-3 (PMC8960818; doi:10.1038/s41420-022-00936-3)
Supplement: Supplementary file 1 — Supplemental text [file 41420_2022_936_MOESM1_ESM.docx]

# SUPPLEMENTAL MATERIALS

## Behavioral Assessments:

Mice were observed daily for evidence of sickness. Any changes in appearance (weight loss, changes in fur), behavior (posture, locomotion, grooming) were monitored. BW was recorded for general toxicity assessment as well as for drug dose adjustment across the treatment schedule.

At baseline as well as post-treatment (24h, 2weeks, and 4weeks) behavioral tests were administered to assess potential neurotoxic effects on locomotor behavior, short-term working memory, mechanical nociceptive thresholds, grip strength, and motor coordination. Tests were performed in order of increasing stress, with a minimum of 20min rest time between sessions. All tests were performed under normal light, between the hours of 10:00 and 14:00 to reduce diurnal effects.

#### Locomotor Open Field Testing

Mice were placed in the center of a rectangular open field chamber (52x33.5x30cm) and allowed to explore freely for 10min. Total distance traveled (cm) and velocity (cm/sec) were recorded for each animal at each of the time points. Mice were visually assessed for abnormal gait or impairment in motor function.

#### Coordination and Balance Assessment – Rotarod Testing

Mice were evaluated for balance and coordination using a Rotarod apparatus (Stoelting Co, Wood Dale, IL). Mice were placed on a rotating drum (beginning speed 4rpm; max speed 12rpm) and evaluated for total time to fall across 3 testing sessions. Session time was limited to 300s to reduce training and endurance effects. Twenty minutes recovery time was allowed between each session, and mice were randomly assigned to testing lanes. The average time across sessions was determined, and the mean values were analyzed across each time point for each animal.

#### Grip Strength Assessment – Wire Grid Hang Testing

Limb strength assessment was measured using a wire grid (37.5x48cm), and mice were placed on the grid as it was slowly inverted 180° and suspended over an empty cage. Mice were allowed to freely explore using both forelimbs and hindlimbs, until unable to grip the wire. The total time before each mouse dropped from the grid was recorded across 3 trials (maximum time 300s), to determine baseline measure. The test was repeated at 3 separate post-treatment time-points, with 3 trials recorded and averaged for each mouse at each testing day, times are expressed as a percent change from baseline score to determine changes post-treatment.

#### Mechanical Nociception – VonFrey Testing

Mechanical thresholds for nociception were used to determine alterations to perceived pain tolerance. Prior to testing, mice were placed into individual Plexiglas chambers (9x11.5x20cm; wire mesh floor) for one hour to acclimatize to the environment. Following acclimatization, VonFrey Filaments of increasing force (2g to 15g) were applied to the plantar surface of the hind paw until visible paw withdrawal, alternating left and right, with a minimum of 2min between each trial. Trials were repeated a total of 3 times for 6 total measurements per mouse. The threshold was expressed as a mean value of 6 measures per animal and was analyzed across time for each animal.

#### Short Term Memory Assessment – Novel Object Recognition

The novel object recognition (NOR) test is an effective mechanism to determine short-term memory deficits in rodents. Mice are placed in the center of the rectangular open field chamber (habituated for 10min, 1 day prior for locomotor assessment) with identical objects attached to the floor with Velcro at either end of the chamber. Mice are allowed free exploration of the chamber and both objects for 10min. They are then returned to their home cage for a 5min delay. One object is removed from the chamber and replaced with a novel object of similar size. Objects are selected to have different shapes, colors, and textures to avoid confusion during testing. Following the 5min delay, mice are returned to the same chamber and allowed 3min to explore. Time spent investigating the familiar object as well as the novel object, total number of approaches to each object, and distance traveled are recorded for each mouse. Time spent is expressed as a discrimination index to identify preferences of familiar or novel in the testing time. Novel object recognition testing is repeated at each time point, with no object being used twice for any individual mouse.

## Cardiovascular Assessments:

Cardiovascular assessments included both standard echocardiogram measurements of cardiac function across time as well as gold-standard endpoint evaluations of hemodynamic function via arterial blood pressure measures and intra-cardiac pressure-volume loops (PV Loops). A particular focus was placed on the evaluation of blood pressure because of previous use of VTD as an anti-hypertensive.

### Echocardiogram

Standard short-axis Motion-Mode (M-mode) echocardiograms using Vevo 2100 (FUJIFILM VisualSonics, Toronto, Canada) ultra-high frequency ultrasound were collected at one-week intervals throughout the four-week VTD treatment. M-mode enables the collection of high-resolution images for precise measurement of cardiac wall and lumen diameters and subsequent calculation of cardiac ejection fraction (EF) using standardized algorithms. EF is a widely accepted hallmark of cardiac function. Additionally, heart rate (HR), cardiac output (CO), and stroke volume (SV) were measured from the echocardiograms.

For echocardiogram collections, mice were rapidly anesthetized with 4% Isoflurane in an induction chamber and maintained at ~1.25% after induction via mask. Body temperature was supported, and heart rate was carefully monitored. Depilatory cream was applied to the anterior chest to remove hair and ultrasound gel was applied to enable ultrasound transducer interface. Standard long and short-axis Brightness-mode (B-mode) and Motion-mode (M-mode) images were captured as video clips using the Vevo 2100 high-frequency ultrasound system for later analysis using Vevo LAB software v5.2.0. For the cardiac function analysis, the short-axis M-mode image was used for the evaluation of heart rate (HR), ejection fraction (EF), stroke volume (SV), and cardiac output (CO).

### Hemodynamic Recordings

At endpoint, animals were evaluated using an SPR-839 PV Loop catheter (Millar, Inc., Houston, TX, USA) to record both arterial and intra-cardiac measures using the PowerLab System and associated LabChart software v8 (ADInstruments, Inc., Colorado Springs, CO, USA). Mice were anesthetized with Isoflurane, orally intubated, and placed on a rodent pressure-controlled ventilator (Microvent1, Hallowell, EMC, Pittsfield, MA, USA) with tidal pressure oscillations set to 5-15mmH_2_O, and 155 breaths per minute. Animals were taped to a heated hard-pad with real-time electrocardiogram to monitor heart rate throughout the surgical procedure (MouseMonitor, Indus Instruments, Webster, TX, USA). The heat pad was set to 40°C and a mylar warming blanket was placed over the animal to maintain body temperature in all animals to ~37°C. Once animals were intubated and placed on the heat pad, they were not manipulated for a full 15min before beginning surgery to ensure optimal body temperature, and thus heart rate, upon hemodynamic catheter arterial placement. After 15min, the skin on the anterior neck was removed, the carotid artery was isolated and retracted rostral with 6-0 silk suture. Another silk suture was placed caudal on the vessel to prepare for ligation around the catheter once inserted. Before introduction of the catheter, a serrifine was placed at the location of the caudal suture, a small hole made for insertion of the catheter, and the catheter advanced up to the serrifine, the serrifine subsequently released, the catheter advanced within the vessel, and secured with the caudal 6-0 suture. The signal was then recorded within the carotid artery of the neck and this recording was utilized for the arterial measurements including arterial mean blood pressure (mBP), systolic blood pressure (sBP), diastolic blood pressure (dBP), and arterial heart rate (HR).

### Pressure-Volume Loops (PV Loop)

PV Loop was used to produce real-time intra-cardiac pressure-volume relationships, and to determine direct intra-cardiac measures of contractility (dp/dt max) and relaxation (dp/dt min). PV Loop assessments were conducted on mice at the end of behavioral, as well as, cardiovascular assessments. Changes in contraction and relaxation of the heart may occur before changes in cardiac wall size that would result in changes in function detectable by echocardiography such as EF. Therefore, to test the full impact of VTD on the heart, we performed gold-standard PV Loops. For PV Loop, some animals were used from both the behavioral and cardiovascular cohorts to bolster animal numbers due to failure rate of PV Loop technique in small-sized mice.

Once carotid arterial measurements were recorded as described above, the catheter was then advanced down the carotid artery, around the aortic arch, and into the left ventricular chamber of the heart. Optimal PV Loop signal was determined by evaluation of the location of the catheter within the heart based on the tallest and widest stable loop signal. The catheter was then stabilized using sutures and taped to the operating table to maintain catheter insertion depth and angle. Immediately following PV intra-cardiac placement, an intravenous (IV) catheter was placed in the left jugular vein and 100µl of 0.9% saline was injected to ensure adequate hydration status. This IV catheter further facilitated administration of the calibration sequence for the PV catheter at procedure end. After both the PV catheter and the IV catheter were placed, animals were left to stabilize for 15min before experimental PV Loop data was recorded. Following the collection of experimental data, calibration steps were conducted per manufacturer’s recommendations (Millar, Houston, TX, USA). Briefly, 20µl of 7.5% hypertonic saline was injected into the IV jugular catheter at three separate intervals and the resultant acute changes in intracardiac pressure and volumes were recorded. This enabled calculation of parallel conductance volume (Vp) shift calculation for volume calibration. Next, administration of 10µl of 1000IU heparin enabled unclotted blood collection for cuvette volume calibration procedure. Finally, the PV Loop catheter was removed, the blood collected from the carotid artery, the chest opened, and the heart excised while still under anesthesia to ensure rapid euthanasia. Data for both arterial measurements and PV Loops were collected and analyzed by an individual blinded to animal treatment.

## Laboratory Methods

### Bioanalysis of VTD in blood/tissues

For bioanalysis of VTD, collected tissue samples were homogenized in water at a concentration of 250 mg/ml prior to analysis. Veratridine concentrations were determined using ultra-high-performance liquid chromatography-tandem mass spectrometry (UPLC-MS/MS) on a Waters Acquity UPLC coupled with a Waters Quattro Premier XE MS/MS (Waters Corporation, Milford, MA, USA). Vinblastine was used as the internal standard for the method. Veratridine was extracted from a 50µl sample aliquot using an acetonitrile protein precipitation approach. After centrifugation, the acetonitrile was decanted into a fresh tube, dried under room air at 40°C, and the sample reconstituted with 60:40 1mM ammonium formate (pH 3.5):methanol prior to injection. Chromatography utilized a Waters Acquity BEH C18, 1.7 µm (2.1 x 50 mm) column with a mobile phases consisting of 5mM ammonium acetate (A) and methanol (B). The mobile phase gradient began at 40% B, which was held for 0.25 min before being linearly increased to 100% B at 2 min. The mobile phase was then held at 100% B for 2 min before being re-equilibrated to the initial conditions prior to the next injection. The mass transition 674.3🡪456.2 (collision energy (CE) 45 eV) was monitored for veratridine (811.4🡪751.4 (CE 40eV) for vinblastine), and the dynamic range of the assay was 0.1 to 500ng/ml (i.e. 0.4 to 2000 pg/mg).

### Cancer Cells for Xenografts

The HCT-116 cell line used for the apoptosis assay was obtained from the ATCC (American Type Culture Collection). HCT-116 iRFP cell line was purchased from Imanis life science (USA). HCT-116 cells were cultured in McCoy’s 5A Medium supplemented with 10% fetal bovine serum and penicillin/streptomycin. Low passages (2 to 5) were used for apoptosis experiments. For the xenograft experiments, HCT-116 iRFP (passage 4) at 70% confluence were incubated with Accutase cell detachment solution (BD Biosciences) for 3min. After centrifugation (2500rpm, 3min), dead cells and debris were removed by two-times wash with ice-cold PBS. Cells were suspended in Hanks’ balanced salt solution without sodium, magnesium, and phenol red (Alfa Aesar, USA) and counted.

### RT-PCR and Western Blot

RNA preparation and quantitative RT-PCR experiments were conducted as previously described^52^. Mouse colon tissues were dissected and cleaned in ice-cold PBS. Following liquid nitrogen freezing, they were stored at -80°C. 60mg of colon tissue was subsequently prepared and placed in a digitonin lysis buffer (50mM Tris/HCl, pH 7.5, 150mM NaCl, 1% Digitonin (Sigma-Aldrich, St. Louis, MO) plus 1x mammalian complete protease inhibitor (Research Products International Corp). Silicon/Zirconia Beads 2.3mm were then added to the tube containing the lysis buffer and cells were mechanically homogenized for 30s with the MiniBead Beater (Biospec Products). Following homogenization, tissue lysates were subjected to centrifugation at 13 000rpm for 10min at 4°C and the supernatant was moved to a fresh Eppendorf tube in preparation for Western Blot (WB). Cell lysates used in WB were normalized for equal loading by NanoDrop using direct absorbance at 280nm (ThermoFisher Scientific). Samples were loaded onto SDS-PAGE 4-20% gradient gel. Protein transfer was performed using an iBlott 2 system for probing with the corresponding antibodies. We used a homemade anti-UBXN2A rabbit polyclonal to detect UBXN2A in tissue lysates (Table 2, Supplemental Materials).^32^ WB membranes were scanned using the LI-COR Odyssey CLx (LI-COR Biosciences, Lincoln, NE, USA) using florescent secondary antibodies and images were evaluated using Image Studio v5.2 software.

### Assessment of apoptosis

HCT-116 cells were treated with different concentrations of VTD (10, 30, 100mM) for 48h. DMSO at a concentration of 0.01% was used as a control. Early and late apoptosis in cells was assessed using a Propidium iodide (PI)/Annexin V Apoptosis Detection Kit (BD Pharmingen) analyzed by a BD Accuri C6 flow cytometer according to the manufacturer’s instructions. Ten thousand gated events were collected per sample. Experiments were conducted in triplicate.

### TUNEL Assay

Paraffin fixed sections of each HCT-116 initiated tumor xenograft were deparaffinized and rehydrated through a graded series of ethanol and water. Slides were then placed in 0.1M citrate buffer pH 6.0 and permeabilized by exposure to 2min 30sec of microwave irradiation (approx. 950W), then 10min at 20% power, and then left in the microwave for 60min in the heated citrate buffer. Staining was performed using a commercially available TUNEL Assay Kit (HRP-DAB-abcam) following the manufacturer’s instructions. Detection was observed as insoluble colored brown signals and studied by a blinded observer (AP) using a light microscope. As previously described, TUNEL staining can label all free 3′-hydroxyl termini regardless of the molecular mechanisms^32^. Based on the timeline of experiments (5 weeks) and the size of terminal tumor masses, we concluded that the stained sections in the studied slides represent a combination of apoptotic and necrotic cells.

# STATISTICAL ANALYSIS

## Behavioral Assessment Statistics

Statistical outliers were identified using separate Grubb’s tests and removed from analysis (2 data points in total). Data analysis was performed by three-way repeated measure ANOVA (treatment x sex x time point) using SPSS software (IBM SPSS Statistics 20). All data for three-way mixed-model ANOVA were adjusted for non-sphericity using a Greenhouse-Geisser correction where necessary, as indicated when degrees of freedom are reported in decimal format (all analyses meeting sphericity have degrees of freedom in whole numbers). Significant interactions in locomotor measures were compared among groups (sex x time point) followed by Student-Newman-Keuls (SNK) when appropriate. Significant effects of timepoint was followed with one-way repeated measures ANOVA and SNK when appropriate. Further ANOVA and post-hoc analyses were performed using SigmaPlot v13.0.

## Cardiovascular Assessment Statistics

Data were analyzed using GraphPad Prism v9 and no outliers were removed in the echocardiogram data. Echocardiogram and hemodynamic data were analyzed using separate t-tests presented as mean ± standard error of the mean (SEM) with an accepted α-level of 0.05. Echocardiogram data investigated across time was analyzed using one-way repeated measures ANOVA. Any potential sex differences were evaluated using two-way ANOVA (treatment x sex).

For the carotid arterial measurements, 8 VTD animals and 8 Control animals were successfully collected at endpoint from the cardiovascular cohort. One outlier in the carotid arterial Control group was identified using the ROUT method^53^ and removed from the analysis. While collection of carotid arterial measurements are less affected by animal size, intracardiac PV Loop typically can only be collected in mice 25g or larger due to the logistics of catheter size relative to left ventricular chamber size. Female mice used for hemodynamics had a mean BW of just 20.9±2.3 at endpoint. Ultimately, only three females were able to be collected for PV Loop measurements with 8 VTD and 4 Control animals collected in total from the two cohorts (cardiovascular and behavioral cohorts). For the VTD group, there were 5 successful males and 3 successful females, and in the Control group 4 successful males and no successful females. We were therefore unable to evaluate sex differences for PV Loop data.

## Xenograft Assessment Statistics

One mouse was identified as an outlier for multiple parameters using the ROUT method in GraphPad Prism v9 and was thus removed from all analyses^53^. Another mouse in the xenograft cohort never developed tumors and was not included in data analysis. Therefore, for analysis, there was an n=12 for Control and n=10 for VTD. 3D ultrasound and fluorescent imaging data were analyzed using separate t-tests and presented as mean ± SEM with an accepted α-level of 0.05. Any potential sex differences were evaluated using two-way ANOVA (treatment x sex).

## All Other Assessment Statistics

All other statistical values presented in this study were analyzed with the software GraphPad Prism 9 and utilized the one-way ANOVA or Student’s t-test. Results are presented as mean ± SEM and α-level was set at 0.05.

Variance was similar between VTD and control groups in the behavioral and cardiovascular cohorts. However, variability within VTD treated groups was higher than control in xenograft cohorts and biological assays. Results from cardiovascular, xenograft, and biological studies are displayed with individual data points to enable detection of variability among animals.

Table 1

| **Effects of Veratridine (VTD) on behavior** | | | | | |
| --- | --- | --- | --- | --- | --- |
|  | **Control Male** | **VTD Male** | **Control Female** | **VTD Female** | **ANOVA** |
|  | **Locomotor Distance (cm)** | | | |  |
| 24 Hours | 3759.18±240.82 | 4450.51±386.19 | 4472.96±177.67 | 4167.27±309.09 | 0.890 |
| 2 Weeks | 4709.34±307.87 | 4463.42±399.90 | 4217.72±317.79 | 3970.31±367.31 |  |
| 4 Weeks | 4274.95±304.03 | 4255.73±537.99 | 3454.39±391.40 | 3754.70±257.71 |  |
|  | **Locomotor Speed (cm/sec)** | | | |  |
| 24 Hours | 6.28±0.40 | 7.42±0.64 | 7.46±0.30 | 6.95±0.52 | 0.893 |
| 2 Weeks | 7.85±0.51 | 7.44±0.67 | 7.03±0.53 | 6.62±0.61 |  |
| 4 Weeks | 7.13±0.51 | 7.09±0.90 | 5.76±0.65 | 6.26±0.43 |  |
|  | **Rotarod (sec)** | | | |  |
| 24 Hours | 285.21±9.72 | 285.54±11.80 | 296.42±2.40 | 290.83±6.66 | 0.521 |
| 2 Weeks | 291.25±8.06 | 292.08±7.92 | 299.71±0.30 | 272.63±19.32 |  |
| 4 Weeks | 298.33±1.67 | 300.00±0.00 | 297.67±2.33 | 291.08±5.84 |  |
|  | **Grid Hang (% Change from Baseline)** | | | |  |
| 24 Hours | 1.55±4.52 | -0.31±5.00 | -0.57±0.95 | 4.30±4.84 | 0.930 |
| 2 Weeks | -7.61±7.98 | -8.41±8.56 | -1.83±2.18 | 5.35±6.23 |  |
| 4 Weeks | -9.33±6.94 | -15.19±7.35 | -0.64±1.02 | -1.28±8.65 |  |
|  | **VonFrey Threshold (g)** | | | |  |
| 24 Hours | 7.80±0.77 | 7.21±0.98 | 7.54±0.87 | 8.04±1.07 | 0.864 |
| 2 Weeks | 8.42±0.99 | 7.73±0.77 | 9.40±1.14 | 10.11±1.50 |  |
| 4 Weeks | 8.77±0.79 | 9.56±1.00 | 9.40±0.69 | 9.90±1.16 |  |
|  | **Novel Object Recognition (Discrimination Index)** | | | |  |
| 24 Hours | 0.62±0.06 | 0.49±0.13 | 0.49±0.09 | 0.66±0.06 | 0.885 |
| 2 Weeks | 0.53±0.05 | 0.65±0.08 | 0.69±0.07 | 0.68±0.08 |  |
| 4 Weeks | 0.72±0.03 | 0.64±0.03 | 0.45±0.21 | 0.57±0.11 |  |

| Name | Manufacturer and Catalog number | Dilution |
| --- | --- | --- |
| Rabbit polyclonal anti-UBXN2A against #C-IQRLQKTASFRELS peptide located in the c-terminus of human UBXN2A | Pacific Immunology Corp | 1:1000 (WB) |
| Anti-Grp75 | Santa Cruz (#sc-133137) | 1:1000 |
| Mouse anti-Glyceraldehyde-3-Phosphate Dehydrogenase antibodies (anti-GAPDH, loading controls and cytoplasmic marker). | Millipore (#ZRB374) | 1:20000 |
| IRDye 800CW Goat anti-Rabbit IgG (H+L), | LI-COR Corporate | 1:3000 |

**Table 2**: This table lists antibodies and their dilutions used in this study.

| Cell line | Company | Checked for |
| --- | --- | --- |
| HCT116-iRFP-Puro | Imanis Life Sciences | This cell line has been tested for mycoplasma contamination and is certified mycoplasma free. |

**Table 3**: This table lists cell lines used in this study.
