# Supplementary figures and images for: Pre-clinical safety and therapeutic efficacy of a plant-based alkaloid in a human colon cancer xenograft model
Source: Cell Death Discov. 2022 Mar 28;8:135. doi: 10.1038/s41420-022-00936-3 (PMC8960818; doi:10.1038/s41420-022-00936-3)

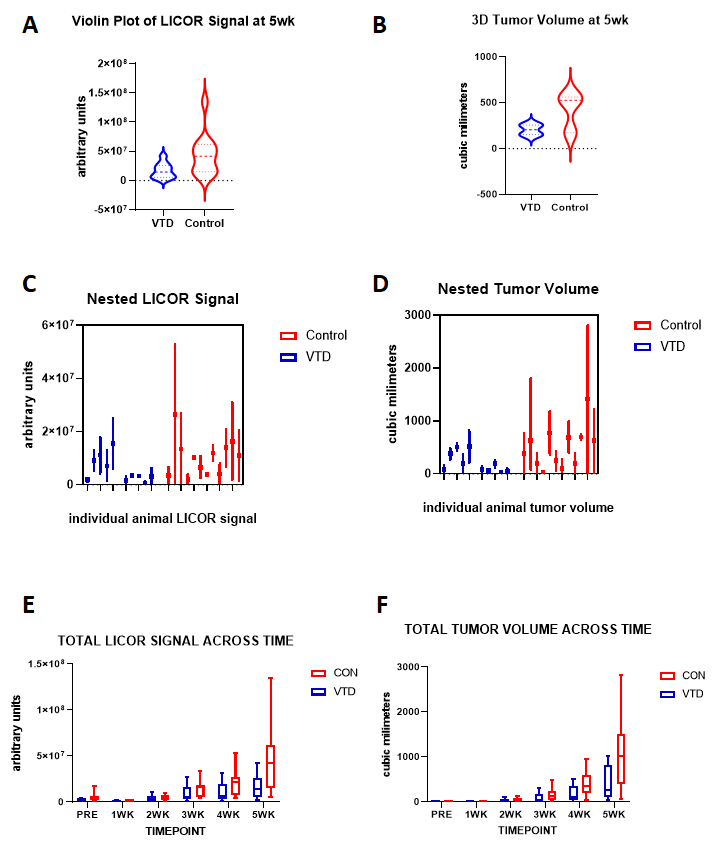

Supplement: Supplementary file 2 — Supplemental Fig. 1 [file 41420_2022_936_MOESM2_ESM.tif]

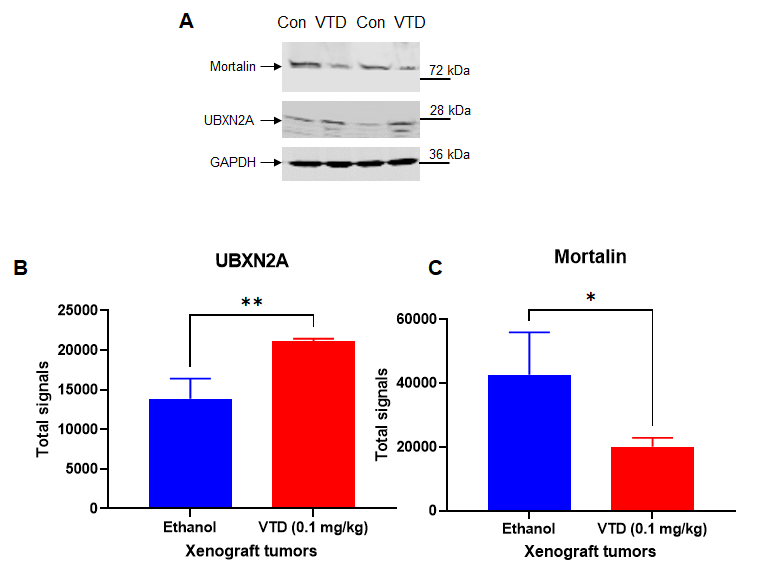

Supplement: Supplementary file 3 — Supplemental Fig. 2 [file 41420_2022_936_MOESM3_ESM.tif]
